# Optimization of heterotrophic cultivation of *Chlorella* sp. HS2 using screening, statistical assessment, and validation

**DOI:** 10.1038/s41598-019-55854-9

**Published:** 2019-12-18

**Authors:** Hee Su Kim, Won-Kun Park, Bongsoo Lee, Gyeongho Seon, William I. Suh, Myounghoon Moon, Yong Keun Chang

**Affiliations:** 1 Department of Chemical and Biomolecular Engineering, Korea Advanced Institute of Science & Technology (KAIST), 291 Daehak-ro, Yuseong-gu, Daejeon 34141 Republic of Korea; 2 Department of Chemistry and Energy Engineering, Sangmyung University, 20 Hongimun 2-gil, Jongno-gu, Seoul 03016 Republic of Korea; 3 Department of Microbial and Nano Materials, College of Science and Technology, Mokwon University, 88 Doanbuk-ro, Seo-gu, Daejeon 35349 Republic of Korea; 4 Advanced Biomass R&D Center, 291 Daehak-ro, Yuseong-gu, Daejeon 34141 Republic of Korea; 5 Gwangju Bio/Energy R&D Center, Korea Institute of Energy Research (KIER), Buk-gu, Gwangju 61003 Republic of Korea

**Keywords:** Biotechnology, Statistical methods, Biodiesel

## Abstract

The heterotrophic cultivation of microalgae has a number of notable advantages, which include allowing high culture density levels as well as enabling the production of biomass in consistent and predictable quantities. In this study, the full potential of *Chlorella* sp. HS2 is explored through optimization of the parameters for its heterotrophic cultivation. First, carbon and nitrogen sources were screened in PhotobioBox. Initial screening using the Plackett-Burman design (PBD) was then adopted and the concentrations of the major nutrients (glucose, sodium nitrate, and dipotassium phosphate) were optimized via response surface methodology (RSM) with a central composite design (CCD). Upon validation of the model via flask-scale cultivation, the optimized BG11 medium was found to result in a three-fold improvement in biomass amounts, from 5.85 to 18.13 g/L, in comparison to a non-optimized BG11 medium containing 72 g/L glucose. Scaling up the cultivation to a 5-L fermenter resulted in a greatly improved biomass concentration of 35.3 g/L owing to more efficient oxygenation of the culture. In addition, phosphorus feeding fermentation was employed in an effort to address early depletion of phosphate, and a maximum biomass concentration of 42.95 g/L was achieved, with biomass productivity of 5.37 g/L/D.

## Introduction

Microalgae have been highlighted as a renewable biomass resource for biofuel over the last few decades due to a number of advantages they have over terrestrial biomass^1^. They are also a promising alternative energy source due to their potentially higher oil content, growth rate, and ability to accumulate high amounts of lipids in comparison to conventional crops and lignocellulosic types of biomass. Today, autotrophic growth is the most common procedure for cultivating microalgae due to their photosynthetic ability and the added benefit of sequestering atmospheric carbon dioxide.

Although microalgae-based biofuels have many advantages, further research and development are necessary to overcome the current problems of low productivity and high cost. The commonly studied process for algal biomass production is outdoor photoautotrophic cultivation, due to the ease of scalability and the added benefit of CO_2_ sequestration. The primary bottleneck that limits the productivity of all photoautotrophic cultivation systems is the light penetration^2^ and absence of light at night^3^. Light penetration is often poor inside photobioreactors (PBR) and open raceway ponds (ORP), and the penetration further falls with increasing cell density and depth due to self-shading effects. Light/dark cycle is also inevitable for outdoor cultivation, and it was reported that respiratory effect at night causes a decrease in the biomass produced in the day period^3^. In addition, the outdoor cultivation systems also suffer from inconsistent biomass productivity due to the fluctuations of light intensity and temperature across the day/night cycle and seasonal changes^4,5^. Furthermore, most common outdoor cultivation systems such as open raceway ponds frequently suffer from contamination problems. Due to the nature of open systems, it is nearly impossible to maintain a monoculture of a single target microalgal strain due to the high susceptibility of these systems to be contaminated with undesirable foreign species^6^. Lastly, concentration of cell culture dictates the costs of harvest, extraction, and purification process of the product, in which case the higher culture density of heterotrophic cultivation provides further advantages of reduced downstream processing costs^7^.

In this light, heterotrophic cultivation has been proposed as an alternative method for industrial-scale algal biomass and biofuel production^1^. One of the major advantages of heterotrophic cultivation is that the culture can achieve more reliable and predictable biomass productivity in comparison to the photoautotrophic systems, due to the consistent availability of the energy source in the form of organic carbon uptake^8,9^. Moreover, additional advantages of heterotrophic cultivation include a cost-effective and relatively simple mode of operation. While additional costs would occur for the usage of carbon sources, there have been many researches which use waste and wastewater containing the massive amount of organic carbon for simultaneously reducing the costs of both algal cultivation and wastewater treatment^8,10,11^. Of course, contamination issues would occur in the outdoor cultivation using organic carbon sources, however, it could be controlled and minimized by using extremophiles^12^. With successful cultivation using the techniques above, it is possible to obtain a high-density culture that is unobtainable under photoautotrophic modes, which allows economic harvesting and easier downstream processing for large-scale production^13^. In some cases, fed-batch cultivation can achieve culture density of over 70 g/L, far exceeding that of the photoautotrophic cultures^8^ and considering that centrifugation which is one of the common harvesting methods brings about 100 g/L cell density, then it surely saves the costs for the harvests^14,15^. Lastly, heterotroph based massive cultivation could make it possible to produce value-added products which have higher prices at least greater more than biofuel^9,16–18^. While it has been estimated that the costs of carbon sources account for up to 80% of the total operating costs in the heterotrophic cultivation, the above-mentioned advantages can easily compensate for the increased costs^19^.

*Chlorella* sp. HS2 was recently isolated from Jeongbang falls in Jeju, Korea. It has the ability to achieve extraordinary culture density (5.91 g/L) and biomass productivity (656.7 mg/L/D) under photoautotrophic conditions^20^. In addition, it is capable of tolerating high-level of salinity (0–5%), and being able to proliferate under a wide range of temperature (14–46 °C) and pH (3.0–10.5)^21^. Due to these outstanding growth and environmental resistance characteristics compared to other microalgae, investigating how this strain would perform under heterotrophic fermentation holds significant interest.

Statistical analysis has been applied on algal biomass and lipid production a lot, because it is an efficient way to optimize complex and various culture conditions including nutrient, temperature, pH and so on^22–24^. However, statistical optimizations were limited to phototrophic cultivation of microalgae, and the application for heterotrophic cultivation has been seldom reported. Most of the previous statistical analyses on heterotrophic cultivation were mainly for producing value-added products instead of focusing on general biomass productivity^25,26^.

Thus, in this study, we optimized the heterotrophic cultivation of promising strain of *Chlorella* sp. HS2 in order to explore the potential of green algae fermentation for biomass and biofuel production using Plackett-Burman design (PBD)^27^ and the response surface methodology (RSM) with the central composite design (CCD)^26,28,29^. The optimized parameters were then experimentally verified on the flask scale and then on the final scale, a 5-L fermenter, for further optimization. Lastly, biomass and lipid productivity was further improved by enhancing the nutrient uptake using a P-feeding strategy.

## Results and Discussion

### Initial screening of the carbon and nitrogen sources

**Figure 1:**
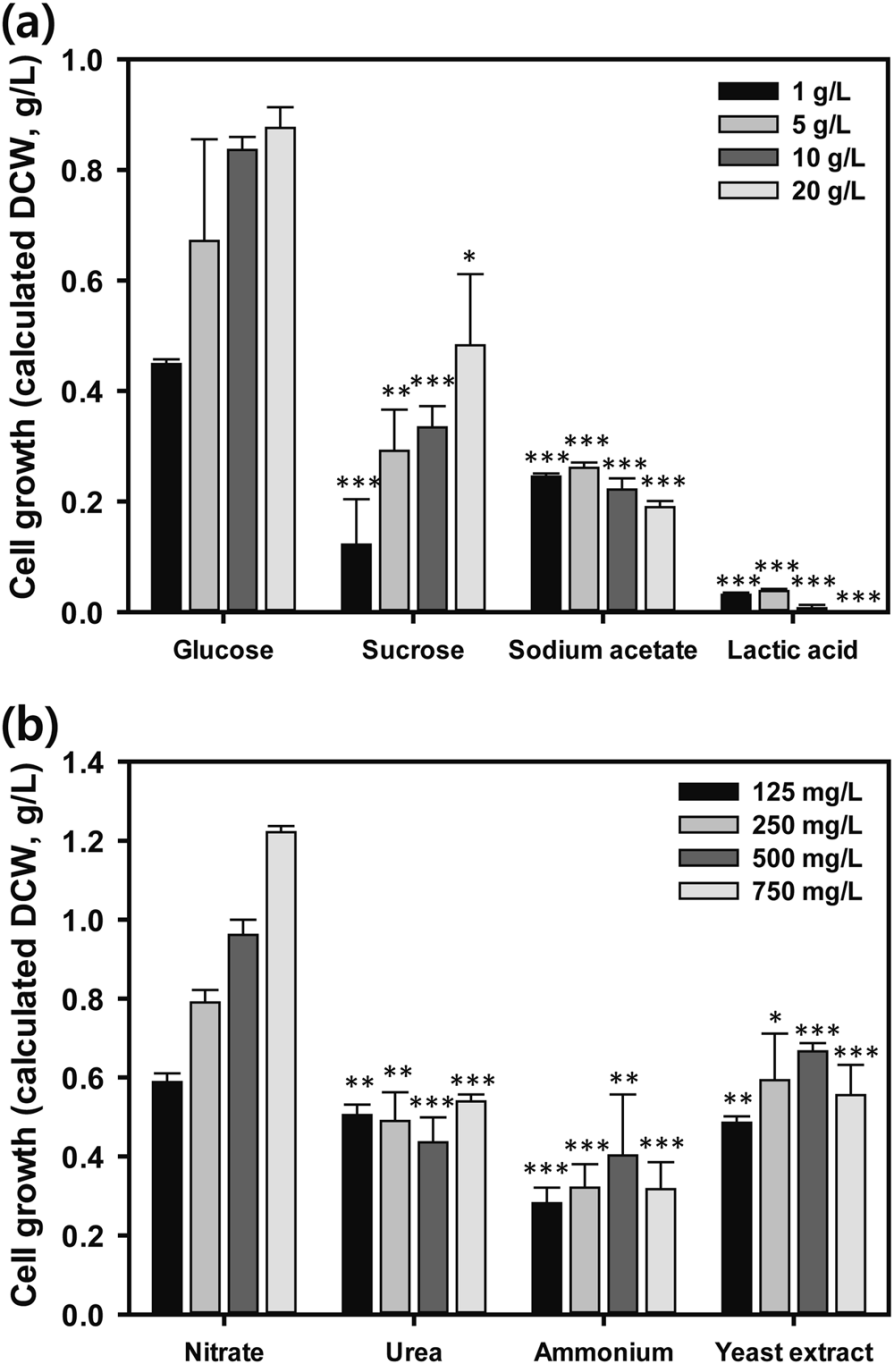
Effects of various concentrations of (**a**) carbon (glucose, sucrose, sodium acetate, and lactic acid) and (**b**) nitrogen sources (sodium nitrate, urea, ammonium chloride, and yeast extract) on the cell growth (calculated dry cell weight, DCW) under heterotrophic cultivation of *Chlorella* sp. HS2. Asterisks (*P <0.05, **P <0.01, ***P <0.001) refers to significant differences when compared with each glucose or nitrate concentrations.

In order to determine the substrate that is most favored by *Chlorella* sp. HS2, glucose, sucrose, sodium acetate, and lactic acid were tested. Glucose was chosen as the candidate because it is the most common substrate in industrial fermentation processes^2,30^. Sucrose is an inexpensive carbon from waste molasses^8^. Acetate and lactate were one of the most common carbon sources for growing many microorganisms, including microalgae^1,2^. Cell growth using four different carbon sources with 1, 5, 10, and 20 g/L concentrations were examined with optical density at 680 nm and it was calculated as dry cell weight (DCW, g/L) using Eq. 2, as presented in Fig. 1a. Among the various concentrations of carbon sources that were tested, the best performance was achieved with 20 g/L of glucose (0.41 g/L), followed by 10 and 5 g/L of glucose (0.39 and 0.36 g/L, respectively). The second best growth performance was observed with sucrose, but the performance was relatively lower than the result with the same concentration of glucose (20 g/L sucrose showed 0.26 g/L). It could be assumed that degradation of sucrose was not processed well because of the neutral pH of the medium^31^ and cells had lower efficiency to use sucrose directly or degraded fructose than glucose. As a result, *Chlorella* sp. HS2 has less capability to use sucrose in this condition and resulted in just half the growth compared to that obtained with the same concentration of glucose. Sodium acetate and lactic acid were the third and fourth carbon source for *Chlorella* sp. HS2. In the case of sodium acetate, it has the ability to buffer the pH^32^, but a high dosage of it could modify the pH to an undesirable range for *Chlorella* sp. HS2. In a similar manner, lactic acid could modify the pH below the optimum range for *Chlorella* sp. HS2 and showed almost no growth. As a result, among the carbon sources, glucose has been utilized as the primary carbon source in microorganism based industries because it is metabolized in the form of energy and a carbon source with minimal cost. *Chlorella* sp. HS2 appeared to easily metabolize glucose and thereupon provide good biomass production. In terms of economic feasibility, glucose is relatively more cost-effective than the other carbon sources because it is produced on a large scale industrially and has a high substrate yield in the cultivation industry^33^. In addition, glucose constitute large part of reasonable carbon sources from agricultural and food waste water^1,34^. Therefore, optimization study based on glucose will help better understand the cultivation using various reasonable carbon sources also^35^. Thus, under these circumstances, glucose was selected as a heterotrophic carbon source for *Chlorella* sp. HS2.

In addition to the carbon source, sodium nitrate, urea, ammonium chloride, and yeast extract were tested for the heterotrophic cultivation of *Chlorella* sp. HS2. Sodium nitrate was chosen as the candidate because it is commonly used as a nitrogen source for *Chlorella* cultivation^36^. Urea is one of the cheapest nitrogen sources and is considered a valuable nitrogen source to achieve high lipid productivity^37^. Ammonium chloride is meanwhile the preferred nitrogen source for most microalgae, as all other forms of nitrogen must be converted to ammonium for accessible nitrogen forms^38^. Finally, yeast extract is an important nitrogen source for all microorganism growth as well as microalgae growth^39^. Each nitrogen source (125, 250, 500, and 750 mg/L) were added to a BG11 medium containing 10 g/L of glucose and cell growth was examined. The results are provided in Fig. 1b. Among the tested conditions, the highest cell growth performance was obtained with 750 mg/L of sodium nitrate (0.58 g/L), whereas 500, 250, and 125 mg/L showed decreasing cell growth (0.46, 0.37, and 0.27 g/L, respectively). Interestingly, the other nitrogen sources showed similar cell growth over different dosages. In the case of ammonium chloride, ammonium ions make hydrogen ions when they are consumed and decrease the pH, and therefore could impede cell growth. In the case of urea, it contains two ammonium ions in the molecules, but it has a smaller effect on pH changes than ammonium chloride^2^. However, different concentrations of dosages did not show significant differences in cell growth and the highest cell growth (0.25 g/L growth with 750 mg/L urea) was even less than the minimum result of sodium nitrate (0.28 g/L growth with 125 mg/L sodium nitrate). Yeast extract contains not only nitrogen source in the form of amino acids but also carbon and phosphorus sources, therefore it usually yields higher cell density than the other nitrogen sources. However, in this screening test, maximum cell growth was achieved under 500 mg/L yeast extract, and further additions reduced the cell growth. Therefore, considering the substrate yield and economic feasibility of nitrogen sources, sodium nitrate was selected for the heterotrophic cultivation of *Chlorella* sp. HS2.

### Screening of key nutrients using the PBD

Based on the results of the carbon and nitrogen screening test described above, glucose and sodium nitrate were selected as the main nutrients for BG11 application on heterotrophic *Chlorella* sp. HS2. In addition, all eleven nutrient sources in glucose added BG11 media with sodium nitrate were evaluated in twelve combinations via PBD^27^. Tables S1 and S2 illustrate the candidate variables (nutrients), the high- and low-end ranges used, and the responses (dry cell weight) under the given conditions on the flask scale. The results summarized in Table 1 show that glucose, sodium nitrate, both phosphate sources, MgSO_4_∙7H_2_O, Na_2_CO_3_, and ferric citrate all positively influenced cell growth. However, CaCl_2_, citric acid, and Na_2_ EDTA were found to have negative effects. Although some of the trace metal solutions displayed a negative effect, the error range is considerably large due to the very low concentrations of the trace metals. Trace metals were not removed from the list of media components given that their presence is still necessary for cell growth. While the choice of phosphorus source did not have a critical effect on the overall cell growth, monopotassium phosphate was observed to have a greater influence on the response (indicated by a higher *t* value) in comparison to dipotassium phosphate. Additional comparison between monopotassium phosphate and dipotassium phosphate was conducted in the flask scale (Fig. S1) because dipotassium phosphate is the only phosphate source in the modified BG11 medium. As a result, it showed no significant difference (P value (0.33)> 0.05) in cell growth. Theoretically, both monopotassium phosphate and dipotassium phosphate were dissociated into dihydrogen phosphate and monohydrogen phosphate, respectively, in the solution and these dihydrogen phosphate and monohydrogen phosphate are in the equilibrium state. And their distribution at the equilibrium state was determined by pH or the other ions in the medium^40^. Though two different monopotassium phosphate and dipotassium phosphate were tested, their pHs were adjusted to pH 7 and the same concentrations of other nutrients were supplied, therefore, the distributions of monohydrogen phosphate and dihydorgeon phosphate ions might be same for both conditions. That is why, we assumed, both phosphate sources showed almost same growth and glucose consumption results as Fig. S1. Therefore, dipotassium phosphate which is usually used in the ordinary BG11 medium was selected. Based on these observations, glucose, sodium nitrate, and dipotassium phosphate were chosen as the carbon, nitrogen, and phosphorus sources, respectively, for the remainder of the study. The minor nutrient components in BG11 were identical to the earlier selections, with the exception of MgSO_4_∙7H_2_O, which was added at a higher concentration (increased from 0.075 g/L to 1 g/L). MgSO_4_∙7H_2_O plays an important role in the formation of the chloroplast of green algae, and therefore MgSO_4_∙7H_2_O was included in the initial RSM. However, when it was present above a certain concentration, MgSO_4_∙7H_2_O was found to not have a remarkable influence on cell growth and was hence excluded as the main factor. Ferric citrate was also shown to have a relatively large effect according to the PBD results, but it had less meaning to find the optimal concentration due to its low concentration in the medium. Therefore, dipotassium phosphate was chosen as a key nutrient for optimization along with glucose and sodium nitrate.

**Table 1:** Effect and statistical analysis of key variable using Plackett-Burman Design.

Variable	Effect	Coefficient	Cell growth (g/L)	
			t-value	p-value
Intercept		1.690	61.46	0.000*
Glucose	2.033	1.017	36.97	0.000*
NaNO_3_	0.800	0.400	14.55	0.000*
K_2_HPO_4_	0.122	0.061	2.22	0.000*
KH_2_PO_4_	0.278	0.139	5.05	0.036*
MgSO_4_∙7H_2_O	0.322	0.161	5.86	0.000*
Na_2_CO_3_	0.156	0.078	2.83	0.000*
CaCl_2_	−0.333	−0.167	−6.06	0.009*
Ferric citrate	0.667	0.333	12.12	0.000*
Citric acid	−0.022	−0.011	−0.40	0.690
Na_2_ EDTA	−0.289	−0.144	−5.25	0.000*
Trace metal solution	−0.600	−0.300	−10.91	0.000*

^*^ Statistically significant at 95% of confidence level; R^2^ = 0.9880.

### Optimization of the medium composition by the RSM

After the initial profiling of the major and minor nutrients, the concentrations of glucose, sodium nitrate, and dipotassium phosphate were optimized by employing the RSM and CCD. For the CCD, a second-order polynomial RSM model consists of 2^3^ factorial design points with six-star points and six replicates at the central points, thus requiring 20 experiments in total^41^. The coded and natural values of the variables are shown in Table S3. After selecting glucose, sodium nitrate, and dipotassium phosphate as the three key nutrients, the CCD was applied to identify the effects of these nutrients on cell growth and thus to determine their optimal concentrations (Table S4). For the determination of the significance, t-tests were conducted, with the p-values presented in Table 2^42^. The interrelationships between the dry cell weight (g/L) and the levels of key nutrients were found to be well represented by the following quadratic equation with a correlation coefficient R^2^ of 0.9050,

1 $${\rm{y}}=15.887+1.565{\rm{G}}-1.953{\rm{N}}+1.931{\rm{P}}-2.709{{\rm{G}}}^{2}-1.33{{\rm{N}}}^{2}-0.747{{\rm{P}}}^{2}-1.513{\rm{GN}},$$

where G, N, and P represent glucose, sodium nitrate, and dipotassium phosphate, respectively.

**Table 2:** Regression coefficients and their significances in CCD and RSM.

Term	Coefficients	*t*-value	*p*-value
Intercept	15.887	22.732	0.000*
G	1.565	3.374	0.007*
N	−1.953	−4.213	0.002*
P	1.931	4.165	0.002*
G^2^	−2.709	−6.001	0.000*
N^2^	−1.330	−2.946	0.015*
P^2^	−0.747	−1.654	0.129
GN	−1.513	−2.496	0.032*
GP	−0.000	−0.000	1.000
NP	−0.000	−0.000	1.000

G: Glucose; N: NaNO_3_; P: K_2_HPO_4_

**Figure 2:**
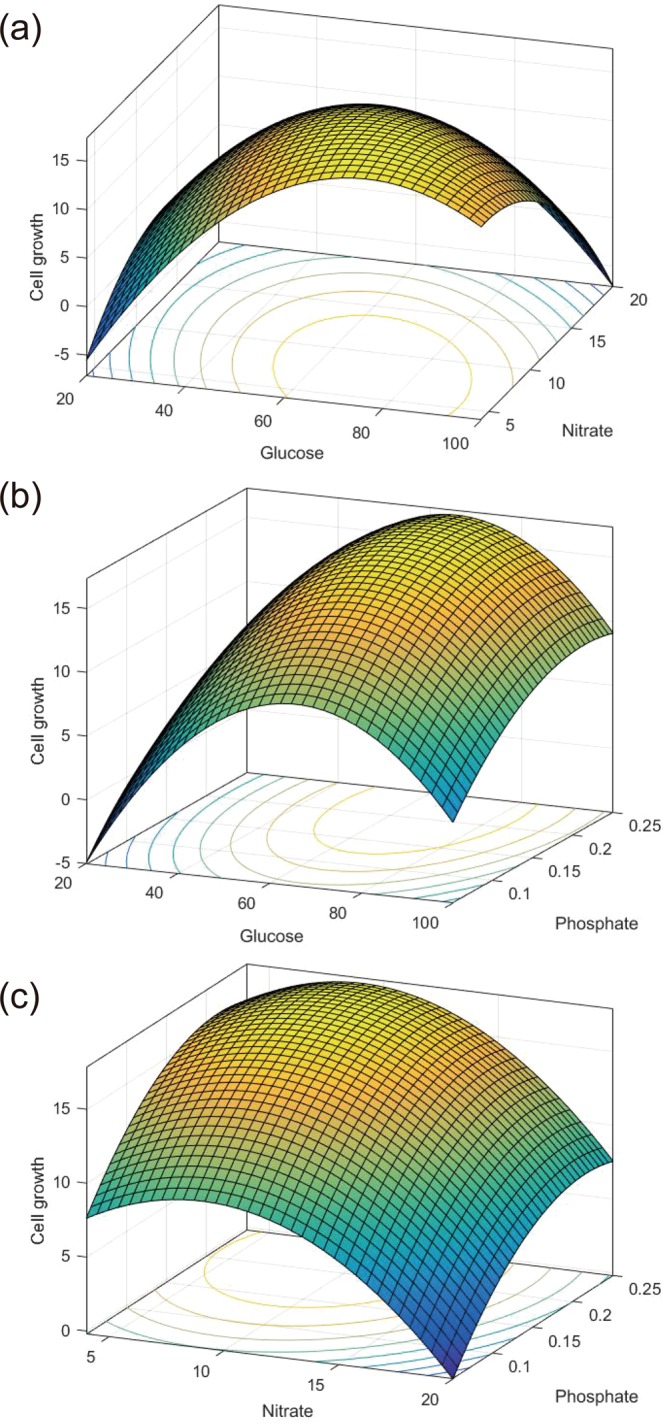
Three-dimensional response surface and contour plots for dry cell weight (g/L) showing the effects of (**a**) glucose and sodium nitrate, (**b**) glucose and dipotassium phosphate, and (**c**) sodium nitrate and dipotassium phosphate concentrations.

Respective contour plots and three-dimensional response surface curves described by the regression model are presented to explain the effects of the key nutrients (Fig. 2). Each response surface curve represents the effect of two of the three nutrient factors, while the third variable is set at the baseline level. The contour plot of G and N appeared to be elliptical, which indicates some degree of mutual interaction among the nutrients. Based on the regression equation, the optimum concentrations were calculated to be 72 g/L glucose, 8 g/L sodium nitrate, and 0.22 g/L dipotassium phosphate.

### Validation of the RSM model via flask cultivation

Next, *Chlorella* sp. HS2 was cultivated under the proposed optimum concentrations of glucose, sodium nitrate, and dipotassium phosphate, which were tested in order to validate the RSM model. Under non-optimized conditions containing 72 g/L glucose in BG11 medium, the maximum biomass of 5.85 g/L was obtained (Fig. S2a). In comparison, according to the model, the maximum dry cell weight (DCW) of the microalgae was predicted to be 18.63 g/L under the optimized conditions. After the microalgae were cultivated for seven days in the optimized medium on the baffled flask, a total DCW of 18.13 g/L was obtained (Fig. S2b). This result was close to the model's prediction of 18.63 g/L.

The accuracy of the RSM model (Eq. 3) was analyzed further by the analysis of variance (ANOVA) approach, and these results are summarized in Table S5. In the flask scale validation, the F-value (F_statistic(9,10)_=10.56) was three times greater than the tabulated F_(9,10)_(=3.14), which shows that the model is a good predictor of the experimental results^43,44^. Furthermore, the relatively high correlation coefficient (R^2^ = 0.9050) and low P-value (P <0.001) provided strong evidence of the high significance of the regression model^45^.

### Optimized fermentation of *Chlorella* sp. HS2 in the 5-L fermenter

The earlier validation experiment on the flask showed that *Chlorella* sp. HS2 cultivated in the optimized BG11 medium presented a remarkable increase in DCW from 5.85 to 18.13 g/L. However, considering that cells were not able to fully utilize all the glucose in the medium (Fig. S2b) and cell growth was limited by the flask scale condition (limited oxygen and changing pH)^46,47^, fermenter scale cultivation should be examined. Therefore, in order to investigate the full potential of *Chlorella* sp. HS2 under heterotrophic conditions, the cultivation was scaled up to a 5-L jar fermenter.

**Figure 3:**
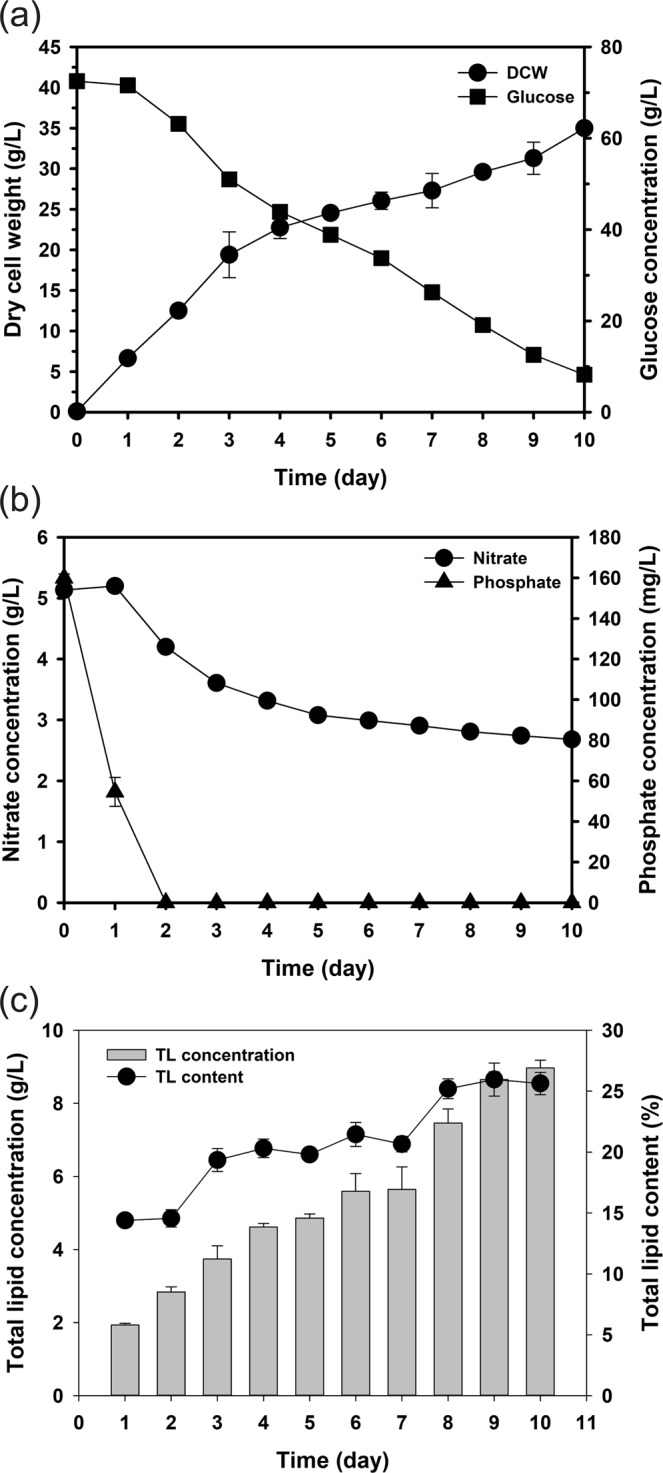
Dry cell weight (DCW), total lipid productivity, and nutrient concentrations throughout the cultivation period under optimum conditions in 5-L fermenter: (**a**) DCW and glucose concentration; (**b**) nitrate and phosphate concentrations; (**c**) total lipid productivity and contents.

As expected, *Chlorella* sp. HS2 in the optimized BG11 medium achieved a maximum DCW of 35.3 ± 0.6 g/L after 10 days with a better glucose consumption rate (6.5 g/L/D) (Fig. 3a). However, even in the fermenter scale, cells failed to use all the glucose. The reason was found in the nutrient profile, as presented in Fig. 3b. Dipotassium phosphate was depleted on the second day and it appears that the depleted phosphate disturbed the cell growth and cell could not utilize all the glucose. According to^48^, the C:N:P ratio is important for harmonized utilization of each carbon, nitrogen, and phosphate source, and it also can be exploited such that the cells achieve greater biomass yield. Even though the concentration of glucose continued to decrease until the end of the experiment, the growth rate of *Chlorella* sp. HS2 was decreased after the third day from 6.47 g/L/D to 2.23 g/L/D, and the cells appear to be in an abnormal condition, showing low activity. In the same manner, the consumption of an optimized amount of sodium nitrate was limited to 2.5 g/L for 10 days (Fig. 3b). Phosphate was depleted quickly and this has a negative effect on nitrate consumption. Limited nitrate consumption caused more lipid accumulation (Fig. 3c). The total lipid productivity continued to decrease throughout the cultivation process due to the increase in the cultivation time. However, the total lipid content increased gradually from 20.2% when the phosphate was depleted to 26.28% on day 8. This increase in the lipid content indicates that the growth was being inhibited by nutrient limitation^37,49,50^. Lipid productivity kept decreasing from 1.42 g/L/D (2^nd^ day) to 0.81 g/L/D (7^th^ day) and subsequently showed a similar value (0.90 g/L/D). This decrease was mainly because cells showed less biomass productivity than changes in lipid content, which showed an increase.

The fermenter scale cultivation gave 1.57 times more biomass yield and 10 g/L higher consumption of glucose. Although screened and optimized conditions were applied, cells could not completely consume the glucose, mainly because of the depletion of phosphate. It is noteworthy that even though screening and statistical optimization were applied, additional optimization is required at a specific scale. Therefore, further optimization was conducted in the form of phosphate feeding.

### P-feeding fermentation for improved carbon-nitrogen utilization

RSM data showed that the optimum dipotassium phosphate feeding amount was 0.22 g/L. In microalgae, phosphates are commonly known to be depleted first due to microalgae quickly uptaking any excess phosphorus in the environment for sequestration in the form of phosphate granules^51^. This phenomenon makes it difficult to precisely determine the amount of phosphate that is used and to control the balance among the C, N, and P sources^48^. In addition, RSM data showed that more addition of dipotassium phosphate led to lower biomass yield, and therefore a phosphate feeding strategy was applied. Although most fed-batch fermentation processes involve the feeding of a carbon source, the P-feeding process is feasible for fed-batch fermentation^52^. The initial medium used was BG11, which is identical to that in the previous experiment, and 1 mL of concentrate containing 0.22 g/L of phosphorus was pulse-fed every two days until the glucose was depleted.

**Figure 4:**
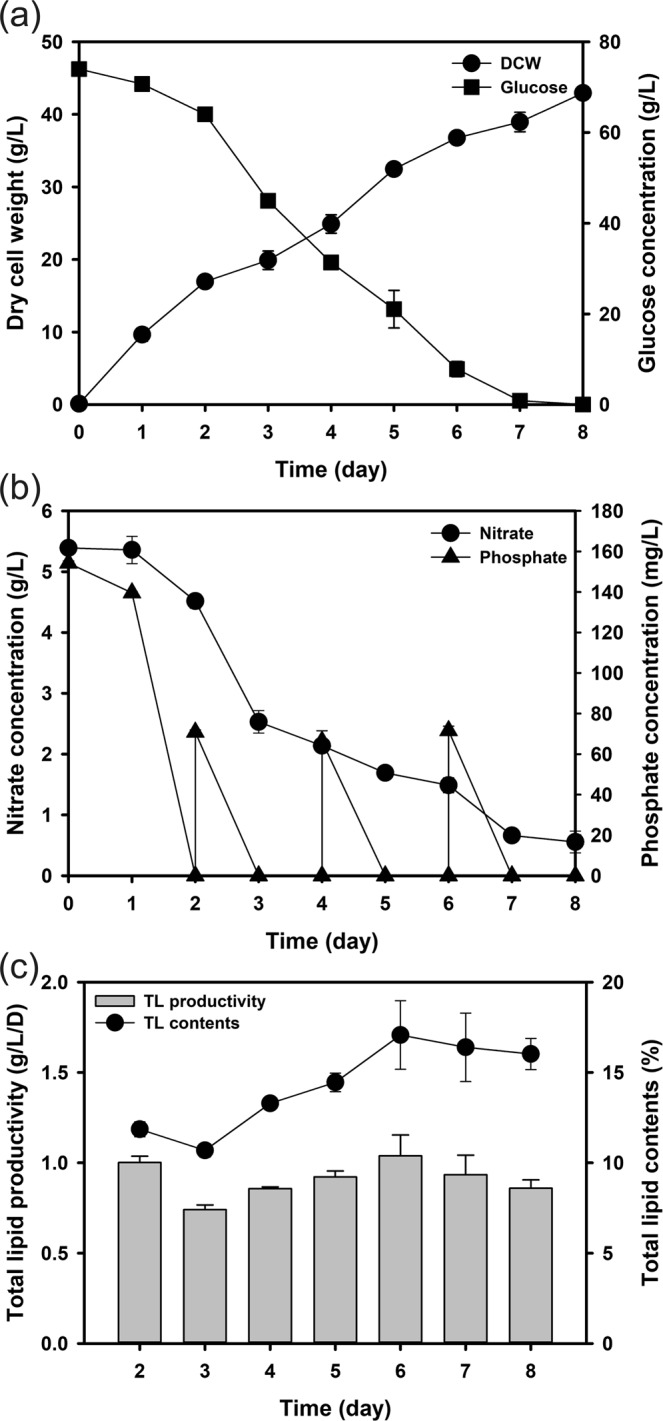
Dry cell weight (DCW), total lipid productivity, and nutrient concentrations throughout the cultivation period under P-feeding process conditions in 5-L fermenter: (**a**) DCW and glucose concentration; (**b**) nitrate and phosphate concentrations; (**c**) total lipid productivity and contents.

The dipotassium phosphate feeding fermentation resulted in similar growth rate (5.37 g/L/D) over the cultivation, as shown in Fig. 4a. The final dry cell weight increased to 42.95 g/L and glucose was completely depleted by the end of the cultivation (8^th^ day) as the glucose consumption rate increased from 6.4 g/L/D (without phosphate feeding) to 9.0 g/L/D (with phosphate feeding). Enhanced glucose utilization could be explained by the fact that greater phosphate availability under pulse-feed culture conditions allowed cells to increase nitrogen uptake (Fig. 4b). All the supplied phosphate with pulse feed was consumed within a day and nitrate consumption was maintained at a constant rate (0.61 g/L/D). Indeed, the total amount of nitrate consumed increased from 2.47 to 4.91 g/L only for 8 days. The previous study also reported that the nitrogen source uptake rate is greatly influenced by the phosphate availability^51^ and it was closely related to the optimum N:P ratio.

By adopting pulse-feeding cultivation of phosphate, the overall dry cell weight and the consumption rates of glucose, nitrate, and phosphate were enhanced. However, better nitrate consumption led to less lipid content during the cultivation (Fig. 4c). The observed decrease most likely resulted from the decrease in the overall C:N ratio within the biomass. While the amount of glucose consumed in the pulse-feeding cultivation of phosphate was slightly greater than that during batch cultivation, and the consumed amount of nitrogen nearly doubled. This may indicate two possible underlying mechanisms; one is easy consumption of the nitrogen source caused by better phosphate availability removes the nitrogen limitation condition from cells and this results in better growth compared to the case of concentrating on the lipid accumulation^48^. The other is that more of the carbon flux was involved in *de novo* protein synthesis and cellular growth than in lipid accumulation (Fig. S3). Through the pulse-feeding cultivation of phosphate, the lipid content increased from 11.86% (day 3) to 16.02% (day 8), although it is substantially lower than cultivation without pulse feed of phosphate (26.28%). As a result, total lipid productivity was also similar to the cultivation without pulse feed of phosphate, but it was kept at around 0.86 g/L/D during the whole cultivation period and this was mostly because of better growth.

The overall performance of biomass production from the system that used *Chlorella* sp. HS2 with the optimized BG11 medium and the P feeding fermentation mode was found to be vastly superior to the performance in the majority of previous works involving the heterotrophic fermentation of green algae (Table 3). In terms of pure DCW yields, the current study achieved the highest biomass concentration among the studies that were surveyed^39,49,53,54^. Most importantly, the biomass yield per substrate input (0.60 g DCW/g substrate) was among the highest ever reported. However, the overall lipid content was found to be noticeably lower (16.0%) than that in other studies (26.3–72.0%).

**Table 3:** Comparison of dry cell weight and lipid content of various *Chlorella* spp. under heterotrophic batch cultivation.

Microalgae	*Chlorella protothecoides* CS-41	*Chlorella protothecoides* UTEX 256	*Chlorella vulgaris* NIES-227	*Chlorella sorokiniana* FC6 IITG	*Chlorella* sp. HS2	*Chlorella* sp. HS2
Substrate	Glucose	Glucose	Glucose	Sodium acetate	Glucose	Glucose
C:N ratio	8.6	30	19.2	17.6	21.8	21.8
Biomass (g/L)	13.9	15.3	4.27	1.75	35.3	43.0
Lipid content (%)	72.0	64.1	30.0	39.2	26.3	16.0
Biomass yield (g DCW/g substrate)	0.46	0.51	0.36	0.12	0.55	0.6
Biomass productivity (g/L/D)	1.39	3.2	0.53	0.11	3.53	5.37
Reference	Li *et al*., ^37^	Chen & Walker, ^54^	Shen *et al*., ^49^	Kumar *et al*., ^39^	In this study (Batch)	In this study (P feed)

It is noteworthy that different C:N ratio within the biomass (based on the consumption) results in different lipid content and biomass yield even within the same system as shown in Figs. 3c and 4c. As expected, the various culture conditions and strains across different studies resulted in a wide range of response (Table 3). While both batch and P feeding cultivation of *Chlorella* sp. HS2 had identical C:N ratio (21.8) in the medium, different biomass yields (35.3 and 43.0 g/L) and lipid contents (26.3 and 16.0 g/L) were obtained. As for two studies involving *Chlorella vulgaris* and *Chlorella sorokiniana*, the C:N ratios in the medium were comparable (19.2 and 17.6 each), but they reported totally different biomass yields (4.27 and 1.75 g/L) and lipid contents (30.0 and 39.2%). In the case of *Chlorella protothecoides*, Li *et al.* and Chen & Walker used different C:N ratio in the medium, but obtained showed similar biomass yields (13.9 and 15.3 g/L) and lipid contents (72.0 and 64.1%). Therefore, it appears that C:N ratio in the medium alone is a poor indicator to predict the biomass and lipid yields, and specific optimization is needed for each target product and selected strain under given conditions. In this study, BG11 medium (originally a phototrophic medium) has been optimized using PBD and RSM with the primary objective of maximizing the rate of biomass productivity. While the lipid content was lower than the values reported in most instances of heterotrophic fermentation, the possibility of increasing the lipid content further can be explored with *Chlorella* sp. HS2. These steps would involve a potential two-stage cultivation mode with nutrient starvation after supplemental glucose fed-batch fermentation, combined with increased delivery of disolved oxygen (DO) for more effective utilization of the supplemental glucose.

## Conclusion

In this study, PBD was employed to determine the effects of various nutrients contained in BG11 on the heterotrophic growth of *Chlorella* sp. HS2. An RSM analysis based on CCD successfully determined the optimal concentrations of glucose, sodium nitrate, and dipotassium phosphate, which were 72 g/L, 8 g/L, and 0.22 g/L, respectively. A subsequent scale-up of the model to a 5-L fermenter showed that the culture underwent possible phosphorus deficiency, resulting in incomplete utilization of the carbon and nitrogen sources. This problem was addressed via P feeding fermentation using pulse-supplementation of phosphorus, which resulted in a maximum biomass concentration of 42.95 ± 0.05 g/L. This led to the conclusion that the heterotrophic cultivation of *Chlorella* sp. HS2 is a highly promising method for the production of high-density algal cultures.

## Materials and Methods

### Microalgal strain and culture maintenance

Green alga *Chlorella* sp. HS2 was isolated by Dr. Hee-Sik Kim at the Korea Research Institute of Bioscience and Biotechnology (KRIBB) in Korea. The culture was maintained in a BG11 medium containing (g/L) 1.5 NaNO_3_, 0.04 K_2_HPO_4_∙2H_2_O, 0.075 MgSO_4_∙7H_2_O, 0.036 CaCl_2_∙2H_2_O, 0.006 citric acid, 0.02 Na_2_CO_3_, 0.006 ferric ammonium citrate, 0.001 Na-EDTA, and 1 mL of the trace metal A5. The trace metal A5 solution consisted of (g/L) 2.86 H_3_BO_3_, 1.81 MnCl_2_∙4H_2_O, 0.22 ZnSO_4_∙7H_2_O, 0.39 NaMoO_4_∙2H_2_O, 0.079 CuSO_4_∙5H_2_O, and 0.05 CoCl_2_∙6H_2_O. The seed culture, which used BG11 medium with 10 g/L glucose, was maintained in a dark condition at 32 °C and 120 rpm.

### Carbon and nitrogen screening using PhotoBiobox

Screening of carbon and nitrogen sources for heterotrophic *Chlorella* sp. HS2 was conducted using PhotoBiobox (Sinhwascience, Korea)^55^. Photobiobox is an incubator invented to keep the same condition (temperature and air supply) for all wells on 96 well plate and it makes possible to compare the effect of certain additives precisely. For the carbon source screening, glucose, sucrose, sodium acetate, and lactic acid were tested. Glucose, sucrose, sodium acetate, and lactic acid, with respective concentrations of 1, 5, 10, and 20 g/L, were added into each 1 L BG11 medium containing 1.5 g sodium nitrate and sterilized with an autoclave. For the nitrogen source screening, sodium nitrate, urea, ammonium chloride, and yeast extract were tested. Each nitrogen source, where the concentrations were 125, 250, 500, and 750 mg/L, was added into each BG11 medium containing 10 g/L glucose and sterilized with an autoclave. Microalgal culture was inoculated in 250 μL wells of a 96-well glass plate (working volume: 200 μL) and it was covered to prevent evaporation^55^. Cells were cultivated for 120 hours under a dark condition at 37 °C and mixed using the auto-mixing function in Photobiobox. Cell growth was initially measured by optical density at 680 nm (OD_680 nm_) using Plate reader (SpectraMax M2; Molecular Devices, Sunnyvale, CA, USA). And dry cell weight (DCW) was calculated based on the predetermined calibration curve using the correlation between dry cell weight and OD_680 nm_, which is represented by Eq. 2.

2 $${\rm{DCW}}=2.0607\,{{\rm{OD}}}_{680{\rm{nm}}}+0.0249,({{\rm{R}}}^{2}=0.9992)$$

For the correlation, cells were cultivated in the flask scale and then OD and DCW were measured three times daily based using plate reader and method below, respectively.

### Statistical optimization

The Plackett-Burman Design (PBD) was used to select the statistically significant factors that are considered to influence the biomass concentration of *Chlorella* sp. HS2^36^. After the selection of three significant factors using the PBD, a factorial response surface methodology (RSM) was utilized to obtain information about the important effects and the interactions between the selected variables with a positive influence on cell growth. In this study, the central composite design (CCD) was employed for the RSM. In RSM, glucose, sodium nitrate, and dipotassium phosphate was used to obtain the respective optimum concentration. Glucose concentration ranges from 25.36 g/L to 94.64 g/L and sodium nitrate concentration ranges from 5.07 g/L to 18.93 g/L and dipotassium phosphate concentration ranges from 0.06 g/L to 0.24 g/L.

### Statistical analysis

During the carbon and nitrogen source selection, P-values were obtained via a Student's t-test between glucose and different carbon sources at the same concentrations, and a Student's t-test between nitrate and different nitrogen sources at the same concentrations. All the experiments were carried out in duplicate. The results were expressed as mean values and standard deviation (SD) between experiments. The analysis of variance (ANOVA) method was used for the statistical analysis of the model. The analysis includes a Fisher test (*F*-test), a related probability P (F) assessment, and the coefficient of determination (*R*^2^), which measures the fit of the regression model. The response surface and contour plots of the model were used to evaluate the interactions between the important variables.

### Validation of heterotrophic cultivation in flask and fermenter

For flask-scale cultivation, the cells were grown in a 250 mL flask using 100 mL of modified BG11, containing 72 g glucose, 8 g NaNO_3_, 0.22 g K_2_HPO_4_∙2H_2_O, 0.075 g MgSO_4_∙7H_2_O, 0.02 g Na_2_CO_3_, 0.006 g ferric ammonium citrate, and 1 mL of the trace metal A5 per 1 L. The trace metal A5 solution consisted of 2.86 g H_3_BO_3_, 1.81 g MnCl_2_∙4H_2_O, 0.22 g ZnSO_4_∙7H_2_O, 0.39 g NaMoO_4_∙2H_2_O, 0.079 g CuSO_4_∙5H_2_O, and 0.05 g CoCl_2_∙6H_2_O per 1 L. For cultivation in a shaking incubator, the temperature and agitation were kept at 37 °C and at 120 rpm, respectively. The initial pH of the medium was adjusted with 1 N HCl solution to 7.0.

For a fermentation test, the cells were grown in a 5-L jar fermenter (CNS Co. Ltd., Korea) containing 3 L of the modified BG11. The filtered air was supplied at a 1.67 vvm flow rate, and temperature and agitation were kept at 37 °C and 300 rpm, respectively. The culture pH was held constant at 7 via the automatic addition of 1 N HCl. The initial concentration of the inoculum was 7.4 ×10^6^ cells/mL, and the total cultivation periods were 10 days (batch) and 8 days (P-feeding). In the P-feeding cultivation, dipotassium phosphate was initially 0.22 g/L and 0.11 g/L per feed.

### Biomass and total lipid analysis

To measure the dry cell weight (DCW), 1 mL of culture was centrifuged at 13,000 rpm for 3 min. The pellets were washed twice with DI water and dried at 90 °C for 24 h in an oven. To measure the total lipid amount, 30 mg of the cell was centrifuged at 4,000 rpm for 10 min. The pellets were washed twice with DI water and stored at −70 °C for two days and lyophilized for three days.

Total lipids were determined using the modified Folch method^56^. The dried cell was grounded into a powder using a bead beater. Thirty mg of biomass was added into 10 mL of chloroform/methanol (2:1, v/v) and sonicated for 4 h. For the phase separation, 2.5 mL of DI water was then added and vortexed for 5 min and centrifuged at 4,000 rpm for 10 min. The lower hydrophobic phase was transferred to another glass vial through an organic solvent filter (PTFE membrane filters 0.2 µm, Sartorius, Germany) using a syringe. Four mL of filtered hydrophobic phase was transferred to a pre-weighed aluminum weighing dish and underwent natural evaporation in the hood. The lipid content was calculated using Eq. 3.

3 $${\rm{Total}}\,{\rm{lipid}}\,({\rm{g}}\,{\rm{of}}\,{\rm{oil}}\,100\,{g}^{-1}\,{\rm{sample}})=\frac{({W}_{L}-{W}_{D})\times {V}_{C}}{{V}_{P}\times {W}_{S}}\times 100$$

W_D_: weight of the aluminum dish, W_L_: weight of aluminum dish with lipid, W_S_: weight of sample, V_C_: volume of chloroform, V_P_: volume of chloroform in the aluminum dish.

### Glucose, nitrate, and phosphate analysis

The sugar concentrations were measured using a high-pressure liquid chromatograph (HPLC, Waters, USA) equipped with a refractive index detector (RI). The Aminex HPX-87H column (300 mm ×7.8 mm; Bio-Rad, USA) was maintained at 65 °C with a mobile phase (0.01 N H_2_SO_4_). The supernatant was passed through a 0.2 μm syringe filter (Sartorius Stedim Biotech, Gottingen, Germany), and the nitrate concentration was measured using ion chromatography (883 Basic IC plus, Metrohm, Switzerland). An anion column (Metrosep A Supp 5) was used to analyze the nitrate (NO_3_^-^) and the phosphate (PO_4_^−^) ions remaining in the culture medium. The eluent, which consisted of 3.2 mM Na_2_CO_3_ and 1 mM NaHCO_3_, was supplied at a flow rate of 0.7 mL/min into the column for the analysis^55,57^.

## Supplementary information

Supplementary tables and figures
