# Supplementary material for: Optimization of heterotrophic cultivation of Chlorella sp. HS2 using screening, statistical assessment, and validation
Source: Sci Rep. 2019 Dec 18;9:19383. doi: 10.1038/s41598-019-55854-9 (PMC6920485; doi:10.1038/s41598-019-55854-9)

**Supplementary Tables and Figures**

**Manuscript number:** SREP-19-25325C

**Title:** Optimization of heterotrophic cultivation of *Chlorella* sp. HS2 using screening, statistical assessment, and validation

**Authors:** Hee Su Kim, Won-Kun Park, Bongsoo Lee, Gyeongho Seon, William I. Suh, Myounghoon Moon, Yong Keun Chang

**Supplementary tables**

Table S1: Levels of the factors tested in Plackett-Burman design.

| Factors | Unit | Experimental value | |
| --- | --- | --- | --- |
|  |  | Low (-1) | High (+1) |
| Glucose | g/L | 1 | 10 |
| NaNO_3_ |  | 0.15 | 1.5 |
| K_2_HPO_4_ |  | 0.039 | 0.39 |
| KH_2_PO_4_ |  | 0.03 | 0.3 |
| MgSO_4_∙7H_2_O |  | 0.075 | 0.75 |
| Na_2_CO_3_ |  | 0.042 | 0.42 |
| CaCl_2_ |  | 0.038 | 0.38 |
| Ferric citrate |  | 0.006 | 0.06 |
| Citric acid |  | 0.006 | 0.06 |
| Na_2_ EDTA |  | 0.001 | 0.01 |
| Trace metal solution | mL / L | 0 | 1 |

Table S2: Plackett-Burman design matrix and responses for initial screening of the nutrients.

| **Run** | **Experimental value** | | | | | | | | | | | **Response** |
| --- | --- | --- | --- | --- | --- | --- | --- | --- | --- | --- | --- | --- |
|  | **G** | **N** | **K2P** | **K1P** | **Mg** | **NC** | **CCl** | **FC** | **CA** | **EDTA** | **TM** | **Cell growth (g/L)** |
| 1 | 1 | -1 | 1 | -1 | -1 | -1 | 1 | 1 | 1 | -1 | 1 | 1.89 |
| 2 | 1 | 1 | -1 | 1 | -1 | -1 | -1 | 1 | 1 | 1 | -1 | 3.59 |
| 3 | -1 | 1 | 1 | -1 | 1 | -1 | -1 | -1 | 1 | 1 | 1 | 0.49 |
| 4 | 1 | -1 | 1 | 1 | -1 | 1 | -1 | -1 | -1 | 1 | 1 | 1.69 |
| 5 | 1 | 1 | -1 | 1 | 1 | -1 | 1 | -1 | -1 | -1 | 1 | 2.69 |
| 6 | 1 | 1 | 1 | -1 | 1 | 1 | -1 | 1 | -1 | -1 | -1 | 3.89 |
| 7 | -1 | 1 | 1 | 1 | -1 | 1 | 1 | -1 | 1 | -1 | -1 | 1.09 |
| 8 | -1 | -1 | 1 | 1 | 1 | -1 | 1 | 1 | -1 | 1 | -1 | 0.79 |
| 9 | -1 | -1 | -1 | 1 | 1 | 1 | -1 | 1 | 1 | -1 | 1 | 1.19 |
| 10 | 1 | -1 | -1 | -1 | 1 | 1 | 1 | -1 | 1 | 1 | -1 | 1.89 |
| 11 | -1 | 1 | -1 | -1 | -1 | 1 | 1 | 1 | -1 | 1 | 1 | 0.69 |
| 12 | -1 | -1 | -1 | -1 | -1 | -1 | -1 | -1 | -1 | -1 | -1 | 0.09 |

G: Glucose; N: NaNO_3_; K2P: K_2_HPO_4_; K1P: KH_2_PO_4_; Mg: MgSO_4_; NC: Na_2_CO_3_; CCl: CaCl_2_; FC: ferric ammonium citrate; CA: citric acid; EDTA: Na-EDTA; TM: trace metal A5 solution

Table S3: Levels of major nutrients as variables in CCD.

| **Variable** | **Code** | **Level (g/L)** | | | | |
| --- | --- | --- | --- | --- | --- | --- |
|  |  | **-1.73** | **-1** | **0** | **1** | **1.73** |
| Glucose | G | 25.36 | 40 | 60 | 80 | 94.64 |
| Nitrate | N | 5.07 | 8 | 12 | 16 | 18.93 |
| Phosphate | P | 0.06 | 0.1 | 0.15 | 0.2 | 0.24 |

G: Glucose; N: NaNO_3_; P: K_2_HPO_4_

Table S4: Twenty planned experimental matrix and results of CCD.

| **Run** | **Coded level** | | | **Cell growth (g/L)** | |
| --- | --- | --- | --- | --- | --- |
|  | **G** | **N** | **P** | **Observed response** | **Predicted response** |
| 1 | -1 | -1 | -1 | 8.95 | 8.05 |
| 2 | 1 | -1 | -1 | 14.29 | 14.20 |
| 3 | -1 | 1 | -1 | 9.51 | 7.17 |
| 4 | 1 | 1 | -1 | 8.80 | 7.27 |
| 5 | -1 | -1 | 1 | 11.97 | 11.91 |
| 6 | 1 | -1 | 1 | 17.31 | 18.06 |
| 7 | -1 | 1 | 1 | 12.53 | 11.03 |
| 8 | 1 | 1 | 1 | 11.82 | 11.13 |
| 9 | -1.73 | 0 | 0 | 3.50 | 5.05 |
| 10 | 1.73 | 0 | 0 | 10.70 | 10.47 |
| 11 | 0 | -1.73 | 0 | 16.00 | 15.28 |
| 12 | 0 | 1.73 | 0 | 6.00 | 8.51 |
| 13 | 0 | 0 | -1.73 | 8.40 | 10.30 |
| 14 | 0 | 0 | 1.73 | 16.90 | 16.99 |
| 15 | 0 | 0 | 0 | 15.90 | 15.89 |
| 16 | 0 | 0 | 0 | 16.00 | 15.89 |
| 17 | 0 | 0 | 0 | 15.94 | 15.89 |
| 18 | 0 | 0 | 0 | 15.98 | 15.89 |
| 19 | 0 | 0 | 0 | 15.93 | 15.89 |
| 20 | 0 | 0 | 0 | 15.96 | 15.89 |

G: Glucose; N: NaNO_3_; P: K_2_HPO_4_

Table S5: Analysis of variance (ANOVA) for RSM model.

| **Source of variation** | **Sum of squares** | **Degree of freedom** | **Mean square** | **F_statistic_ (MSR/MSE)** | **Prob. (P)** |
| --- | --- | --- | --- | --- | --- |
| Regressions | 279.066 (SSR) | 9 | 31.007 (MSR=SSR/d.f.) | 10.56 | 0.001 |
| Error | 29.366 (SSE) | 10 | 2.927 (MSE=SSE/d.f.) |  |  |
| Total | 308.432 (SST) |  |  |  |  |

Determination coefficient R^2^=0.9050

**Supplementary figures**

Figure S1: Comparison test between (a) dipotassium phosphate and (b) monopotassium phosphate in flask cultivation. Modified BG11 was containing 10 g glucose, 1.5 g NaNO_3_, 0.075 g MgSO_4_∙7H_2_O, 0.02 g Na_2_CO_3_, 0.006 g ferric ammonium citrate, and 1 ml of the trace metal A5 per 1 L. 100 mL working volume in 250 ml baffled flask, 120 rpm was applied.

(a)

(b)

Figure S2: the results of flask cultivation with (a) non-optimized and (b) optimized medium

(a)

**
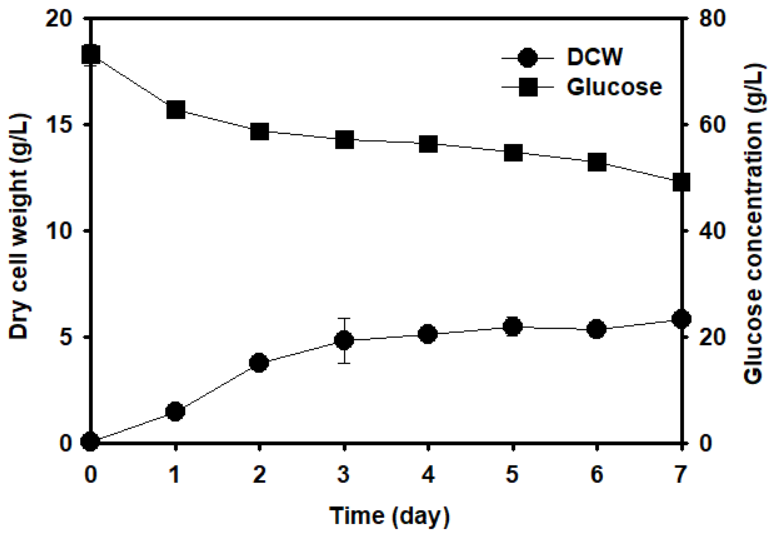
**

(b)


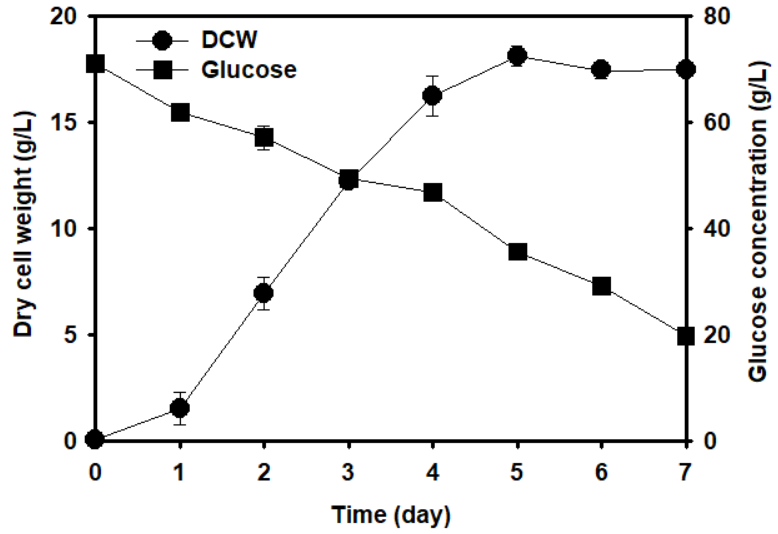


Figure S3: Biomass composition under batch mode and P-feeding cultivation


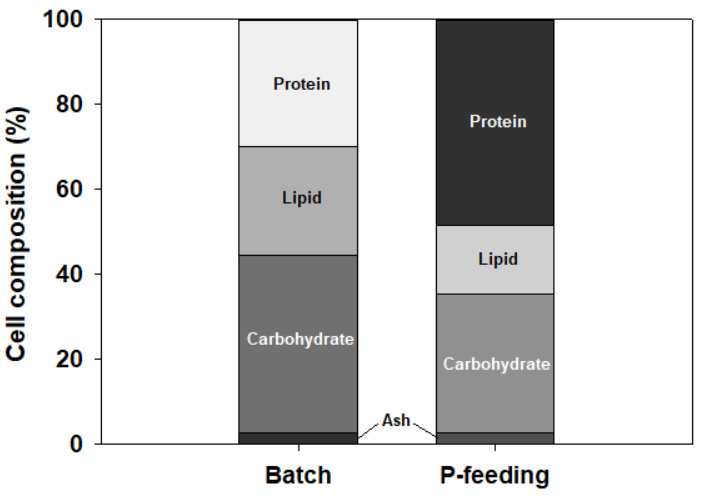

Supplement: Supplementary file 1 — Supplementary tables and figures [file 41598_2019_55854_MOESM1_ESM.docx]
